# The Obesity Paradox in Real-World Nation-Wide Cohort of Patients Admitted for a Stroke in the U.S.

**DOI:** 10.3390/jcm11061678

**Published:** 2022-03-17

**Authors:** Guy Rozen, Gabby Elbaz-Greener, Gilad Margolis, Ibrahim Marai, Edwin K. Heist, Jeremy N. Ruskin, Shemy Carasso, Ariel Roguin, Edo Y. Birati, Offer Amir

**Affiliations:** 1 Division of Cardiovascular Medicine, Hillel Yaffe Medical Center, Hadera 38100, Israel; gilad.margolis@gmail.com (G.M.); arielr@hy.health.gov.il (A.R.); 2 The Ruth and Bruce Rappaport Faculty of Medicine, Technion, Haifa 3109601, Israel; 3 Cardiac Arrhythmia Service, Massachusetts General Hospital, Boston, MA 02114, USA; kheist@mgh.harvard.edu (E.K.H.); jruskin@mgh.harvard.edu (J.N.R.); 4 Department of Cardiology, Hadassah Medical Center, Jerusalem 9574401, Israel; gabbyelbaz100@gmail.com (G.E.-G.); oamir@hadassah.org.il (O.A.); 5 Faculty of Medicine, Hebrew University of Jerusalem, Jerusalem 91240, Israel; 6 The Lydia and Carol Kittner, Lea and Benjamin Davidai Division of Cardiovascular Medicine and Surgery, Baruch Padeh Medical Center, Poriya 1528001, Israel; IMarai@poria.health.gov.il (I.M.); shemy.carasso@gmail.com (S.C.); EBirati@poria.health.gov.il (E.Y.B.); 7 The Azrieli Faculty of Medicine in the Galilee, Bar-Ilan University, Safed 1311502, Israel

**Keywords:** body mass index, BMI, sudden cardiac death, obesity paradox

## Abstract

Background: Obesity has been associated with increased incidence and severity of various cardiovascular risk factors and increased risk for stroke. However, the evidence of its effect on outcomes in stroke victims have been equivocal. We aimed to investigate the distribution of BMI in a nation-wide cohort of individuals, admitted for a stroke, and the relationship between BMI and in-hospital mortality. Methods: Data from the U.S. National Inpatient Sample (NIS) was collected, to identify hospitalizations for stroke, between October 2015 and December 2016. The patients were sub-divided into six groups based on their BMI: underweight, normal weight, overweight, obese I, obese II and extremely obese groups. Various sociodemographic and clinical parameters were gathered, and incidence of mortality and the length of hospital stay were analyzed. Multivariable analysis was performed to identify independent predictors of in-hospital mortality. Results: A weighted total of 84,185 hospitalizations for stroke were included in the analysis. The approximate mean patients aged was 65.5 ± 31 years, the majority being female (55.3%) and white (63.1%). The overall in-hospital mortality during the study period was 3.6%. A reverse J-shaped relationship between the body mass index and in-hospital mortality was documented, while patients with elevated BMI showed significantly lower in-hospital mortality compared to the underweight and normal weight study participants, 2.8% vs. 7.4%, respectively, *p* < 0.001. Age and several comorbidities, as well as the Deyo Comorbidity Index, were found to predict mortality in a multivariable analysis. Conclusion: A reverse J-shaped relationship between body mass index and in-hospital mortality was documented in patients admitted for a stroke in the U.S. during the study period. The above findings support the existence of an “obesity paradox” in patients hospitalized following a stroke, similar to that described in other cardiovascular conditions.

## 1. Introduction

Constantly growing number of people are affected by obesity worldwide. According to a recent report by the World Health Organization, the rate of obesity has tripled since the 70s, and by 2016 over 1.9 billion adults (31%) were overweight, of them 650 million (13%) were obese [1]. The prevalence of obesity in the U.S. has increased significantly in the last few decades, leading to significant social, clinical and economic implications [2,3,4,5,6].

Obesity and stroke, both reaching epidemic proportions, are clearly associated. Based on recent data, up to 25% of adults over the age of 25 are expected to experience a stroke during their lifetime [7]. Worldwide, stroke is a leading cause of long-term disability [8] and is responsible for 5.8 million death cases yearly [9]. Obesity is also a well-established risk factor for cerebrovascular disease, and the body-mass-index (BMI) has a nearly linear association with the risk for ischemic stroke [10,11,12,13,14]. 

Numerous studies linked obesity with higher incidence and increased severity of several cardiovascular risk factors, including increased risk for stroke. However, studies looking into the effect of obesity on outcomes in stroke victims have produced conflicting results. Several studies showed favorable outcomes, in terms of mortality, in overweight and obese stroke patients [15,16,17,18,19], including in those who suffered from a hemorrhagic stroke [20,21,22], supporting the theory of an “obesity paradox” in these patients. However, other studies have shown a detrimental effect of obesity on post-stroke longevity [23], and identified age as a modifying factor with higher mortality risk in younger obese stroke survivors and a lower risk in older ones [24]. As the impact of obesity on stroke outcomes is still under debate, the aim of this study was to describe the patient characteristics and BMI distribution in a nation-wide cohort of patients, admitted for stroke in the U.S., and the association between BMI and mortality as well as length of hospital stay.

## 2. Methods

A detailed description of the study methods and the used dataset has been provided previously by our group [25]. 

### 2.1. Data Source

The data were collected from the National Inpatient Sample (NIS), the Healthcare Cost and Utilization Project (HCUP), and Agency for Healthcare Research and Quality (AHRQ) [26,27]. The NIS dataset represents an approximate 20% sample of all inpatient hospitalizations in the U.S. [28]. The database includes various patient- and hospital-level characteristics such as patient demographics, discharge diagnoses and procedural diagnoses, comorbidities and length of hospital stay (LOS). Hospital level factors include the hospital geographic region, teaching status, and bed size. National estimates are calculated using the patient-level sampling weights, provided by the HCUP. 

For this study, we collected data for U.S. hospitalizations between October 2015 and December 2016. The ICD-10 coding system (International Classification of Diseases, 10^th^ Revision, Clinical Modification—ICD-10-CM) was used from October of 2015 and thereafter to report procedural and clinical diagnoses in the NIS database. The reason we included only the data coded with ICD-10 system is that the ICD-10 codes include individual codes for BMI values and ranges. 

### 2.2. Study Population and Variables

We included patients aged 18 years and above, with a primary diagnosis of a stroke based on one of the I63.XXX ICD-10-CM codes, who had a BMI code documentation in the secondary diagnoses. As we have described our group’s prior publication [25], we have categorized the study population into six BMI subgroups: using BMI ≤ 19 kg/m^2^, the under-weight group; BMI 20–25 kg/m^2^, normal-weight group; BMI 26–30 kg/m^2^, over-weight group; BMI 31–35 kg/m^2^, obese I group; BMI 36–39 kg/m^2^, obese II group; BMI ≥40 kg/m^2^, extremely obese group.

Different demographic parameters were obtained from the NIS dataset, including: age, sex, and race. Prior comorbidities were identified utilizing the appropriate ICD-10 codes among the secondary diagnoses in the dataset. Deyo-Charlson Comorbidity Index (Deyo-CCI) was calculated for each patient, using the 17 comorbidity conditions [29] (Appendix A Table A1 includes all the Deyo-CCI codes). The index has been used extensively in clinical studies, utilizing administrative databases, with good prognostic validity in regards to short- and long-term outcomes [30,31]. Of notice, we used age groups in the analysis, instead of using age as a continuous variable since all the patients aged 90 and above are listed as 90 years old in the database. We wanted to include the entire population of patients with stroke, not excluding the nonagenarians, hence we were forced to use age groups for accurate statistical analysis. We did include the “approximate mean age” in the results section and the abstract, however all the statistical analyses were performed with age groups for improved accuracy. The primary outcome in this study was in-hospital mortality. Length of hospital stay was the secondary outcome analyzed.

### 2.3. Statistical Analysis

We used the Wilcoxon Rank Sum test and chi-square (χ^2^) test and to compare continuous and categorical variables, respectively. The NIS dataset includes discharge sample weights, calculated within each sampling stratum as the ratio of discharges in the universe to discharges in the sample [32]. We have created a weighted logistic regression model to identify independent predictors of mortality during the hospitalization. Candidate variables included various patient clinical and demographic characteristics, Deyo-CCI and hospital-level factors. Variables, associated with primary and secondary outcomes were included in our final multivariable regression model. For all statistical analyses, we utilized SAS^®^ software version 9.4 (SAS Institute Inc., Cary, NC, USA). A *p*-value < 0.05 was considered statistically significant. 

## 3. Results

### 3.1. Study Cohort

A total of 16,837 hospitalizations for stoke in the U.S. were included in the analysis. After implementation of the weighting method, this hospitalizations sample represented an estimated total of 84,185 hospitalizations for stroke. There was a female predominance in the study population (55.3%), and the approximate mean age was 65.5 ± 31 years.

The majority of the study population (63.1%) was white, 56.9% had Medicare coverage (Table 1). With regards to the clinical characteristics, 38% had history of diabetes mellitus, 67% had essential hypertension, 10% had peripheral vascular disease and 23% had a history of atrial fibrillation or flutter. The median BMI in the study was 34 (IQR: 29–41), while 82.4% of the patients had BMI above normal (>25 kg/m^2^).

### 3.2. Patients’ Characteristics and in-Hospital Outcomes in the Different BMI Groups

Table 1 includes the baseline characteristics of the study cohort, divided into the six predefined BMI subgroups. The BMI distribution varied significantly within the different income percentiles and country regions (*p* < 0.001). Female predominance was noted in the underweight and in patients with BMI ≥ 40 (extremely obese). Younger age and increased prevalence of diabetes, hypertension and heart failure were observed in the obese patient subgroups (Table 1).

Of the total population admitted for a stroke, 8.7% received thrombolytic therapy. Obese patients had higher chances to receive thrombolytic therapy during the hospitalization compared to underweight and normal weight patients, 9.3% vs. 6.2%, respectively (*p* < 0.001). Only 2.8% of the total study population underwent mechanical thrombectomy and 1.3% underwent in hospital carotid endarterectomy.

**Figure 1:**
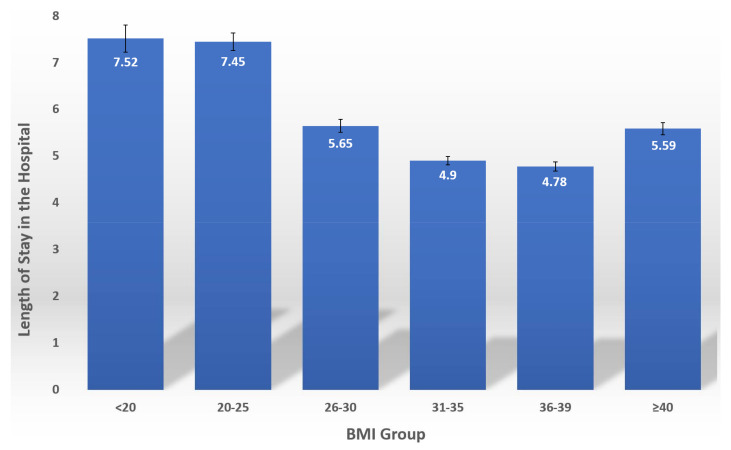
The relationship between BMI and the length of hospitalization in the study.

The average length of stay in the hospital was 5.64 ± 0.07 days. Figure 1 shows the relationship between BMI and the length of hospitalization in the study population. The correlation between BMI and LOS was a reverse J shape in nature with shorter hospital stay in overweight and obese patients, *p* < 0.001.

**Figure 2:**
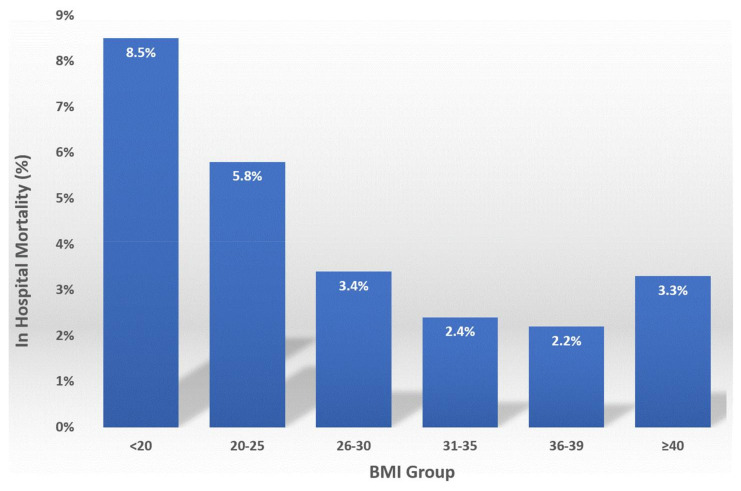
The relationship between BMI and in-hospital mortality in the study.

The rate of in-hospital mortality in patients hospitalized with a stroke was documented at 3.6%. Figure 2 shows the correlation between BMI and in-hospital mortality in the study population. A reverse J-shaped relationship between the BMI and the in-hospital mortality was observed, with higher mortality in underweight and normal weight patients, *p* < 0.001.

### 3.3. Predictors of in-Hospital Mortality

Several parameters were found to significantly increase the odds of in-hospital mortality in an unadjusted analysis (Table 2). These included: age, white race, personal history of hypertension, renal failure, peripheral vascular disease, heart failure and atrial fibrillation/flutter (all with *p* < 0.01). Higher BMI as well as diabetes were associated with reduced mortality in a univariate analysis.

After adjusting for potential confounders, most of the above (except peripheral vascular disease) remained an independent predictor of in-hospital mortality in a multivariable analysis (Table 3). Higher BMI and diabetes were independent predictors of improved survival. Deyo Comorbidity index of ≥2 was also an independent predictor of mortality in patients hospitalized for stroke, OR-2.39 (1.94–2.94), *p* < 0.001.

## 4. Discussion

Using the NIS database, we analyzed a weighted total of 84,185 hospitalizations for a stroke, during the study period of Oct. 2015 to Dec. 2016. This nation-wide data analysis showed a reverse J-shaped association between the BMI and mortality in hospital, in the study population. Elevated BMI was found to be an independent predictor of lower in-hospital mortality and length of hospital stay in patients admitted for a stroke during the study period.

In this study, we aimed to analyze the relationship between BMI and in-hospital outcomes in a nation-wide population of patients, admitted in the US following a cerebrovascular event, in order to overcome the potential biases of the prior, smaller studies. In addition, we wanted to improve the differentiation between the different obese patients BMI subgroups, dividing them into mild (30 < BMI ≤ 35), moderate (35 < BMI < 40) and extremely obese (BMI ≥ 40) groups. The different sociodemographic and clinical characteristics of our study population were comparable with prior publications on stroke patients as to their age and comorbidities such as hypertension, diabetes mellitus, atrial fibrillation and others [16,19,24].

A recent systematic review of 25 studies (299,750 patients) showed that a vast majority of these studies reported a survival benefit for obese study participants, suffering from a stroke. However, due to methodological limitations, study design, lacking adjustment for comorbidities, selection bias and survival bias, it was difficult to draw firm conclusions as to the real-world relationship between BMI and outcomes in post stroke patients [9].

The effect of elevated BMI on the risk for stroke can be explained by elevated prevalence of risk factors such as diabetes, hyperlipidemia and hypertension in the obese patients. However, prospective studies did not show that these co-morbidities necessarily modify the relationship between BMI and stroke [11,12]. In our study increased BMI was documented to have an independent, positive effect on the mortality, despite an almost doubling of the diabetes prevalence in patients with elevated BMI, when compared to the underweight and normal weight patients. The positive effect of the BMI possibly drove the counterintuitive correlation between diabetes and improved survival, documented in this study OR-0.76 (0.69–0.83), *p* < 0.001.

The data presented here reveals a reverse J-shaped relationship between the body mass index and in-hospital mortality in stroke patients, while the mild and moderately obese patient subgroups (BMI 31–39) exhibited the lowest in-hospital mortality in this study. Prior studies have suggested that the “obesity paradox”, described in various cardiovascular conditions including in myocardial infarction, heart failure and sudden death, may apply to patients admitted with a stroke, resulting in lower mortality in obese patients [15,16,17,18,19]. Importantly, many of the prior investigations clustered all patients with BMI > 30 into one study group, showing improved outcomes. We have further subdivided the obese patients in three groups, as described in the methods section. We documented decreased mortality only in the first two of these subgroups (30 < BMI ≤ 35 and 35 < BMI < 40), while in extremely obese patients (BMI ≥ 40), the documented mortality was higher than in patients with BMI between 31–39 kg/m^2^ (Figure 2).

Interestingly, patients suffering from overweight and obesity were shown to be younger, a fact that could have theoretically contributed to better survival in these patients. Niedziela et al, has described this observation in the past, showing that in 20 out of 26 reports included in his meta-analysis of the “obesity paradox” in myocardial infarction studies, patients suffering from overweight and obesity were younger compared to patients in other subgroups [33].

Several protective pathophysiological mechanisms have been suggested to play a role in improving the survival of critically ill patients, including the potential benefit of nutritional reserves in obese patients [34,35,36] and neurohormonal mechanisms linked to higher leptin levels in obese individuals [37,38,39]. We assume that similar mechanisms may have played a role in stroke patients who are exposed to an intense continuous metabolic stress associated with the acute cerebrovascular event and hospitalization. As expected, patients with higher body mass index suffered from high prevalence of cardiovascular risk factors such as HTN and DM. It seems that the adverse effects of these cardiovascular risk factors on patients’ outcomes are counterbalanced by the protective mechanisms that improve the survival in obese individuals hospitalized with a stroke, many of whom suffer from HTN and DM.

This implication of obesity and improved survival after a stroke should be by no means interpreted as supporting weight gain. Elevated body weight index has been documented to be an independent risk factor for several cardiovascular conditions such as ischemic heart disease, myocardial infarction, heart failure, cardiac arrhythmia (both atrial and ventricular), sudden cardiac arrest and stroke [35,40]. The evidence in favor of preventive strategies to fight obesity is unequivocal and our findings should not be misinterpreted as suggesting otherwise.

Our study has several limitations. The National Inpatient Sample database is an administrative retrospective database, containing discharge-level records, hence, it is susceptible to potential coding errors. The lack of patient identifiers in the NIS database does not allow us to analyze other outcomes such as 30-day mortality. We could only document events occurring during the hospitalization, with no longer term follow up. We also cannot rule out that some of the hospitalizations were “readmissions” of the same patients with another cerebrovascular event. Several important parameters are not collected into the NIS database such as additional patient characteristics, chronic medical therapy, laboratory tests etc. Therefore, we cannot completely rule out residual confounding of the correlation we observed in this study. These limitations are counterbalanced by the nation-wide, real-world nature of the data, lack of selection bias and the absence of reporting bias introduced by selective publication from specialized centers.

In conclusion, a reverse J-shaped correlation between the body mass index and in-hospital mortality was documented in patients hospitalized for a stroke in the U.S. during this study period. The presented findings support the existence of an “obesity paradox” in patients hospitalized following a stroke, similar to that described in other cardiovascular conditions.

## Figures and Tables

**Table 1:** Baseline Characteristics of the Study Population (total and per BMI groups).

	Total	<20	20–25	26–30	31–35	36–39	≥40	*p*-Value
BMI, *n*								
Unweighted	16,837	1745	1217	2307	4032	2721	4815	
Weighted	84,185	8725	6085	11,535	20,160	13,605	24,075	
Age Group, %								<0.001
18–44 years	7	2	2	4.8	6.5	9.3	11	
45–59 years	26	9	11	20	27	31.8	34	
60–74 years	39	28	31	39.7	42	41.4	41	
≥75 years	28	61	57	35.8	24.1	17.5	14	
Gender, %								<0.001
Female	55	63	53	48.2	48.9	51.4	64	
Male	45	37	47	52	51	48.5	36	
Missing	0	0	0	0.3	0.1	0	0	
Race, %								<0.001
White	63	67	61	62.1	62.9	63.3	63	
Non-white	31	27	31	32	31	31.3	31	
Other/Missing	6	6	8	5.7	6.2	5.4	6	
Hypertension	67	58	60	65.5	70.2	70	68	<0.001
Congestive Heart Failure	15	16	15	13	13	13.7	17	<0.001
Diabetes Mellitus	38	15	25	36.1	40.5	42.4	46	<0.001
Renal Failure	20	20	21	22	18	18.9	20	<0.001
Chronic Pulmonary Disease	20	26	17	15.7	16.5	19.7	22	<0.001
Peripheral Vascular Disorders	10	15	12	11.9	10	8.9	8	<0.001
Atrial Fibrillation/Flutter	23	31	31	23	20	20.3	22	<0.001
Prior MI	7.9	7.7	7.5	8.2	8.5	7.6	7.5	0.002
VT/VF	1.7	2.2	2.1	1.7	1.7	1.8	1.4	<0.001
Deyo-CCI, %								<0.001
1	8.4	7.6	6.2	7.9	9.1	9	8.6	
2 or higher	91.6	92.4	93.8	92.1	90.9	91	91.4	
Primary Payer, %								<0.001
Medicare	57	80	74	62.1	52.7	49	50	
Private insurance	24	8	12	22	27	30.1	29	
Medicaid	12	8	9	9.5	12.1	12.7	14	
Self-pay	4	2	3	4	5.4	5.1	5	
No charge	0	0	0	0.4	0.3	0.5	0	
Other/Missing	3	2	3	2	3	2.5	3	
Income Percentile, %								<0.001
0 to 25th percentile	33	32	33	31.7	32.1	33.5	35	
26th to 50th percentile	26	24	24	25	27	25.5	26	
51st to 75th percentile	23	21	23	23.5	22.6	23.5	23	
76th to 100th percentile	17	21	19	18.1	16.5	16.3	14	
Missing	2	1	2	2	1.6	1.2	2	
Hospital Status, %								<0.001
Urban teaching	68	67	68	68.1	66.4	67.1	69	
Urban non-teaching	25	24	26	25.3	26.9	25.9	25	
Rural	7	9	7	7	7	6.9	7	
Hospital Region, %								<0.001
South	42.3	40.5	42.4	43.9	43	40.9	42.3	
Midwest	23.7	21.4	22.7	21.2	24	24.4	25.4	
West	17.9	19.3	19.5	20.3	18.3	17.4	15.8	
Northeast	16.1	18.9	15.4	14.6	14.8	17.3	16.5	
Hospital Bed Size, %								0.033
Large	56	57	56	56.2	55.5	56.1	57	
Small/Medium	44	43	44	43.8	44.5	43.9	43	

*p*-values were generated using Chi-square test and refer to differences between BMI groups within baseline characteristics.

**Table 2:** Univariate Analysis for Predictors of In-Hospital Mortality.

Predictor	Probability (95% CI)	Odds Ratio (95% CI)	*p*-Value
Age Group, years			<0.001
18–44 years	2.09% (1.75, 2.48)	1.00 (reference)	n/A
45–59 years	2.15% (1.96, 2.35)	1.03 (0.84, 1.26)	0.771
60–74 years	3.51% (3.32, 3.72)	1.71 (1.42, 2.06)	<0.001
≥75 years	5.59% (5.31, 5.90)	2.78 (2.31, 3.35)	<0.001
Race			<0.001
Non-white	3.14% (2.94, 3.36)	1.00 (reference)	n/A
White	3.81% (3.65, 3.98)	1.22 (1.12, 1.33)	<0.001
Gender			0.546
Male	3.59% (3.41, 3.79)	1.00 (reference)	n/A
Female	3.67% (3.51, 3.85)	1.02 (0.95, 1.10)	0.546
BMI Group			<0.001
20–25	5.75% (5.19, 6.37)	1.00 (reference)	n/A
Below 20	8.54% (7.97, 9.14)	1.53 (1.34, 1.74)	<0.001
26–30	3.38% (3.07, 3.73)	0.57 (0.49, 0.66)	<0.001
31–35	2.38% (2.18, 2.60)	0.40 (0.35, 0.46)	<0.001
36–39	2.21% (1.97, 2.47)	0.37 (0.32, 0.43)	<0.001
40 and Above	3.30% (3.08, 3.54)	0.56 (0.49, 0.64)	<0.001
Atrial Fibrillation/Flutter			<0.001
No	2.78% (2.65, 2.91)	1.00 (reference)	n/A
Yes	6.50% (6.16, 6.86)	2.43 (2.26, 2.62)	<0.001
Chronic pulmonary disease			<0.001
No	3.39% (3.26, 3.53)	1.00 (reference)	n/A
Yes	4.64% (4.33, 4.97)	1.39 (1.27, 1.51)	<0.001
Congestive heart failure			<0.001
No	3.39% (3.26, 3.52)	1.00 (reference)	n/A
Yes	5.07% (4.70, 5.47)	1.52 (1.39, 1.67)	<0.001
Diabetes Mellitus			<0.001
No	4.05% (3.89, 4.23)	1.00 (reference)	n/A
Yes	2.95% (2.77, 3.14)	0.72 (0.67, 0.78)	<0.001
Hypertension			<0.001
No	5.06% (4.81, 5.33)	1.00 (reference)	n/A
Yes	2.92% (2.79, 3.06)	0.56 (0.52, 0.61)	<0.001
Peripheral vascular disorders			<0.001
No	3.52% (3.40, 3.66)	1.00 (reference)	n/A
Yes	4.61% (4.19, 5.08)	1.32 (1.19, 1.47)	<0.001
Renal failure			<0.001
No	3.29% (3.16, 3.42)	1.00 (reference)	n/A
Yes	5.06% (4.73, 5.40)	1.57 (1.44, 1.70)	<0.001
Deyo-CCI			<0.001
1	1.48% (1.23, 1.79)	1.00 (reference)	n/A
2 or higher	3.83% (3.70, 3.97)	2.65 (2.18, 3.22)	<0.001
Income Percentile			0.342
0 to 25th percentile	3.68% (3.46, 3.90)	1.00 (reference)	n/A
51st to 75th percentile	3.68% (3.42, 3.96)	1.00 (0.91, 1.10)	0.977
26th to 50th percentile	3.58% (3.34, 3.83)	0.97 (0.88, 1.07)	0.565
76th to 100th percentile	3.35% (3.07, 3.66)	0.91 (0.81, 1.02)	0.092

**Table 3:** Multivariable Analysis for Predictors of In-Hospital Mortality.

Predictor	Probability (95% CI)	Odds Ratio (95% CI)	*p*-Value
Age Group, years			<0.001
18–44 years	1.41% (1.14, 1.74)	1.00 (reference)	n/A
45–59 years	1.32% (1.14, 1.53)	0.93 (0.76, 1.15)	0.514
60–74 years	1.95% (1.71, 2.21)	1.39 (1.14, 1.69)	0.001
≥75 years	2.67% (2.35, 3.04)	1.92 (1.57, 2.35)	<0.001
Gender			0.023
Male	1.85% (1.62, 2.10)	1.00 (reference)	n/A
Female	1.69% (1.48, 1.92)	0.91 (0.84, 0.99)	0.023
Race			<0.001
Non-white	1.63% (1.42, 1.87)	1.00 (reference)	n/A
White	1.91% (1.69, 2.16)	1.18 (1.08, 1.29)	<0.001
BMI Group			<0.001
20–25	2.55% (2.17, 3.00)	1.00 (reference)	n/A
Below 20	3.85% (3.35, 4.43)	1.53 (1.34, 1.75)	<0.001
26–30	1.44% (1.23, 1.69)	0.56 (0.48, 0.65)	<0.001
31–35	1.14% (0.98, 1.32)	0.44 (0.38, 0.51)	<0.001
36–39	1.07% (0.90, 1.26)	0.41 (0.35, 0.49)	<0.001
40 and Above	1.73% (1.51, 1.99)	0.67 (0.58, 0.78)	<0.001
Atrial Fibrillation/Flutter			<0.001
No	1.58% (1.39, 1.79)	1.00 (reference)	n/A
Yes	2.91% (2.53, 3.33)	1.86 (1.71, 2.02)	<0.001
Congestive heart failure			<0.001
No	1.73% (1.53, 1.96)	1.00 (reference)	n/A
Yes	2.20% (1.90, 2.56)	1.28 (1.16, 1.41)	<0.001
Chronic pulmonary disease			0.002
No	1.75% (1.54, 1.98)	1.00 (reference)	n/A
Yes	2.00% (1.73, 2.32)	1.15 (1.05, 1.26)	0.002
Diabetes Mellitus			<0.001
No	1.84% (1.63, 2.08)	1.00 (reference)	n/A
Yes	1.40% (1.21, 1.62)	0.76 (0.69, 0.83)	<0.001
Hypertension			<0.001
No	2.27% (1.99, 2.59)	1.00 (reference)	n/A
Yes	1.53% (1.34, 1.73)	0.67 (0.62, 0.72)	<0.001
Obesity			<0.001
No	3.15% (2.76, 3.61)	1.00 (reference)	n/A
Yes	1.11% (0.96, 1.27)	0.34 (0.31, 0.39)	<0.001
Peripheral vascular disorders			0.392
No	1.76% (1.56, 1.99)	1.00 (reference)	n/A
Yes	1.85% (1.57, 2.18)	1.05 (0.94, 1.18)	0.392
Renal failure			<0.001
No	1.71% (1.51, 1.93)	1.00 (reference)	n/A
Yes	2.32% (2.01, 2.68)	1.37 (1.25, 1.49)	<0.001
Income Percentile			0.011
0 to 25th percentile	1.86% (1.63, 2.12)	1.00 (reference)	n/A
26th to 50th percentile	1.86% (1.62, 2.13)	1.00 (0.90, 1.11)	0.995
51st to 75th percentile	1.81% (1.57, 2.09)	0.97 (0.88, 1.08)	0.608
76th to 100th percentile	1.55% (1.33, 1.81)	0.83 (0.74, 0.94)	0.002
Deyo-CCI			<0.001
1	1.15% (0.93, 1.42)	1.00 (reference)	n/A
2 or higher	2.70% (2.48, 2.93)	2.39 (1.94, 2.94)	<0.001

## Data Availability

The Healthcare Cost and Utilization Project (HCUP) Data Use Agreement doesn’t allow us to make the data from the NIS database, used for this study, available to other. The NIS database is available for purchase by the public and our detailed and transparent description of methods used for the data analysis allows anyone who wishes to do so to reproduce our results.

## Appendix A

**Table A1:** ICD-10 codes used in the study.

Medical Condition.	ICD-10 CM Codes	Score
Myocardial infarction	I22.xx, I21.xx, I25.2	1
Congestive heart failure	I09.9, I11.0, I13.0, I13.2, I25.5, I42.0, I42.5-I42.9, I43.x, I50.x, P29.0	1
Peripheral vascular disease	I70.x, I71.x, I73.1, I73.8, I73.9, I77.1, I79.0, I79.2, K55.1, K55.8, K55.9, Z95.8, Z95.9	1
Cerebrovascular disease	G45.x, G46.x, H34.0, I60.x-I69.x	1
Dementia	F00.x-F03.x, F05.1, G30.x, G31.1	1
Chronic pulmonary disease	I27.8, I27.9, J40.x-J47.x, J60.x-J67.x, J68.4, J70.1, J70.3	1
Rheumatologic disease	M05.x, M06.x, M31.5, M32.x-M34.x, M35.1, M35.3, M36.0	1
Peptic ulcer disease	K25.x-K28.x	1
Mild liver disease	B18.x, K70.0-K70.3, K70.9, K71.3-K71.5, K71.7, K73.x, K74.x, K76.0, K76.2-K76.4, K76.8, K76.9, Z94.4	1
Diabetes	E10.0, E10.l, E10.6, E10.8, E10.9, E11.0, E11.1, E11.6, E11.8, E11.9, E12.0, E12.1, E12.6, E12.8, E12.9, E13.0, E13.1, E13.6, E13.8, E13.9, E14.0, E14.1, E14.6, E14.8, E14.9	1
Diabetes with chronic complications	E10.2-E10.5, E10.7, E11.2-E11.5, E11.7, E12.2-E12.5, E12.7, E13.2-E13.5, E13.7, E14.2-E14.5, E14.7	2
Hemiplegia or paraplegia	G04.1, G11.4, G80.1, G80.2, G81.x, G82.x, G83.0-G83.4, G83.9	2
Renal disease	I12.0, I13.1, N03.2-N03.7, N05.2-N05.7, N18.x, N19.x, N25.0, Z49.0-Z49.2, Z94.0, Z99.2	2
Any malignancy including leukemia and lymphoma	C00.x-C26.x, C30.x-C34.x, C37.x-C41.x, C43.x, C45.x-C58.x, C60.x-C76.x, C81.x-C85.x, C88.x, C90.x-C97.x	2
Moderate or severe liver disease	I85.0, I85.9, I86.4, I98.2, K70.4, K71.1, K72.1, K72.9, K76.5, K76.6, K76.7	3
Metastatic solid tumor	C77.x-C80.x	6
Acquired Immunodeficiency syndrome (AIDS)	B20.x-B22.x, B24.x	6
